# Placental Histiocyte Phenotypes in Chronic Histiocytic Intervillositis: A Comprehensive Immunophenotypic and Morphologic Atlas

**DOI:** 10.3390/ijms27073024

**Published:** 2026-03-26

**Authors:** Elise Gradhand, Luisa Strahler, Julia Bein, Margarete Mijatovic, Hannah-Ida Hullmeine, Andreas Weigert, Stephan Spahn, Eva Herrmann, Franz Bahlmann, Ella Hullmeine

**Affiliations:** 1 Dr. Senckenberg Institute of Pathology, University Hospital Frankfurt, 60596 Frankfurt am Main, Germanyella.hullmeine@gmx.de (E.H.); 2 Institute of Biochemistry I, Faculty of Medicine, Goethe-University Frankfurt, 60629 Frankfurt am Main, Germany; 3 Institute of Psychology, Humboldt-University Berlin, 10117 Berlin, Germany; 4 Department for Immunity of Inflammation, Mannheim Institute for Innate Immunoscience (MI3), Medical Faculty Mannheim, Heidelberg University, 69117 Mannheim, Germany; 5 Department of Obstetrics and Gynecology, Buergerhospital—Dr. Senckenberg Foundation, 60318 Frankfurt am Main, Germany; 6 Institute of Biostatistics and Mathematical Modelling, Goethe University Frankfurt, 60629 Frankfurt am Main, Germany

**Keywords:** placenta, chronic histiocytic villitis, macrophages

## Abstract

Chronic histiocytic intervillositis (CHI) is a placental lesion characterized by an inflammatory response, significantly influencing maternal and fetal outcomes. This study aims to develop a comprehensive morphologic atlas detailing the localization of fetal and maternal macrophages within the context of CHI. We employed immunohistochemical and multiplexing techniques to analyze placental samples, identifying expression patterns and spatial distribution of key macrophage markers, including CD68, CD163, CD14, and HLA-DR. The results revealed a marked accumulation of activated macrophages in both the intervillous space and villous stroma, with distinct differences in morphology and immunophenotype of fetal Hofbauer cells versus maternal macrophages. Our findings contribute to a better understanding of the immune landscape in CHI and provide a valuable resource for further research into placental immune dynamics. By establishing this morphologic atlas, we aim to enhance diagnostic and therapeutic strategies for affected pregnancies, thereby improving the diagnostic approach and making it more straightforward to recognize CHI histologically.

## 1. Introduction

The human placenta is characterized by two distinct macrophage populations: Hofbauer cells (HBCs) of fetal origin and placenta-associated maternal macrophages (PAMMs). HBCs are first identified within the mesenchymal connective tissue of the villous stroma during the first trimester. As gestation progresses, HBCs migrate within the mesenchyme, displaying pleomorphic morphology [1,2,3]. They express several macrophage-specific markers but notably lack human leukocyte antigen DR (HLA-DR) expression during the first trimester, which only begins to appear in minimal amounts during the second trimester [4,5]. This expression pattern suggests possible origins from erythro-myeloid precursor cells in the yolk sac or primitive macrophage precursors within the placenta itself [1].

Functionally, HBCs are critical for early placental vasculogenesis and angiogenesis, secreting factors such as vascular endothelial growth factor, osteopontin, and sprouty protein [6,7,8]. They also play a role in tissue remodeling and the clearance of cellular debris, neutralizing potentially harmful immune complexes and influencing placental function and regenerative capacity. Their immune response may be more focused on preventing local inflammation than on broad immune defense, showing a regulated response to pathogens, particularly those in the STORCH group (e.g., Syphilis, toxoplasmosis, and rubella), influenced by the chemokine microenvironment [1,9,10,11,12].

In contrast, PAMMs derive from maternal monocytes and arrive at the placenta via the maternal bloodstream, differentiating upon exposure to chemotactic signals from the placental villi [13]. PAMMs are crucial not only for immune defense but also for placental regeneration, secreting matrix metalloproteinases and fibronectin in response to inflammation [7,14]. Their activation can lead to beneficial regenerative processes or harmful inflammatory responses, such as in cases of intervillositis characterized by maternal-derived mononuclear cell infiltration [1].

Chronic histiocytic intervillositis (CHI) is histologically identified by the accumulation of CD68+ macrophages, predominantly of maternal origin, alongside increased fibrin deposition. Being clinically significant despite its low incidence, CHI is associated with complications such as intrauterine growth restriction and recurrent miscarriages [15]. Defined as an infiltrate of CD68+ macrophages occupying at least 5% of the intervillous space, CHI presents with accompanying populations of CD4+ and CD8+ T-lymphocytes and variable fibrin deposition. While less prevalent than villitis of unknown etiology (VUE), CHI’s clinical course is often more severe [16,17,18].

The pathogenesis of CHI may involve an immunological component reacting to placental paternal alloantigens, with elevated levels of TGFBR1 and MMP-9 observed in affected cases [19]. Additionally, the expression of CD200, an anti-inflammatory immune checkpoint, could inhibit macrophage proliferation [20,21]. This suggests a complex interaction between immune tolerance mechanisms and inflammatory responses in placentation [16,22].

VUE, on the other hand, is an inflammatory lesion with a higher incidence [23,24] and is believed to be associated with autoimmune mechanisms. Maternal-derived immune cells, particularly T-lymphocytes, infiltrate fetal tissue, contributing to its pathological effects. Hofbauer cells express immune markers such as HLA I and II, facilitating their involvement in immune responses and antigen presentation [25,26,27].

Interestingly, both CHI and VUE may represent two ends of a continuum of placental inflammation, with studies indicating that macrophage mitotic activity contributes to the inflammatory landscape in VUE [25,28,29]. The lack of identifiable infectious causes in both conditions points to complex immunological interactions, potentially resembling graft-versus-host disease [30,31,32].

Moreover, the impact of SARS-CoV-2 on pregnancy during the pandemic raised concerns regarding placental changes and vertical transmission [33,34,35,36]. Although initial studies suggested heightened risks for pregnant women, subsequent research indicated no increased risk of adverse outcomes [37]. Nonetheless, the potential association between SARS-CoV-2 and placental chronicity, particularly in cases with histological features resembling CHI, warrants further exploration [38,39,40].

This study aims to immunohistochemically phenotype the inflammatory cells in CHI to aid their identification and improve routine pathological assessments. CHI is diagnosed exclusively through histopathological examination, as it lacks specific clinical symptoms or significant sonographic findings. It also seeks to localize macrophage populations within the placenta, enhancing our understanding of fetal and maternal immunological dynamics. Additionally, the study will compare the immunological profiles of placentas from maternal SARS-CoV-2 infections with those from CHI cases to identify any specific inflammatory patterns associated with the virus. Analysis will involve various immunohistochemical markers and multiple labeling immunofluorescence techniques to detail cell localization in healthy and pathological placentas.

## 2. Results

### 2.1. Clinical Characteristics of the Cohort

**Figure 1:**
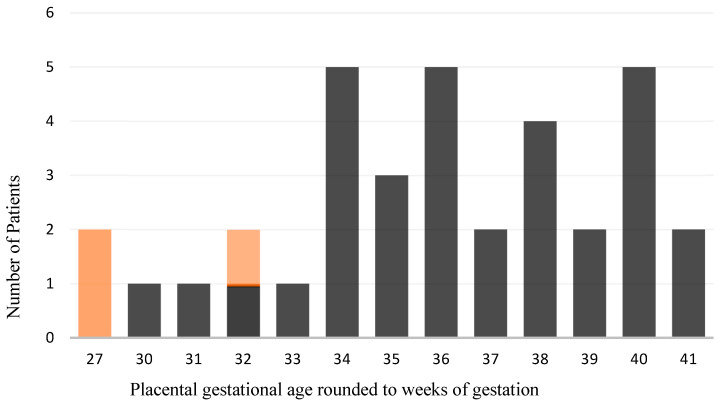
Distribution of weeks of gestation in the CHI/VUE cohort combined with the CHI/SARS-CoV-2 cohort (highlighted in orange).

It was noted that CHI and VUE occurred only in the second and third trimesters (see Figure 1).

### 2.2. Histopathological Findings

#### 2.2.1. The Findings of Marker Expression Within the Individual Compartments

The cohort was analyzed with respect to the localization of immunohistochemically positive immune cells within the groups (see Table 1). A distinct expression pattern was elucidated for each immunohistochemical marker assessed. A finding was classified as positive when at least one cell exhibited reactivity for the respective antigen within the examined section.

Upon examination of the results, it became apparent that the expression patterns of individual markers exhibited significant differences between the CHI/VUE cohort and the control group (Table 1).

The expression pattern observed in the control group was as follows: In all 23 samples, CD3-positive T-lymphocytes were consistently identified in both the villous and intervillous spaces, whereas CD4- and CD8-positive T-lymphocytes were undetectable in healthy parenchyma. The dendritic cell marker CD11c was variably detected in one sample from the intervillous space. CD14-positive cells were consistently located in the villous stroma, with a substantial proportion of samples also exhibiting positivity in the intervillous space. Furthermore, there was a notable heterogeneous distribution of CD68- and CD163-positive cells; the former was consistently identified in the villous stroma and occasionally in the intervillous space, while the latter was predominantly found in the villous stroma, with only one sample demonstrating CD163-positive monocytes in the intervillous space.

In stark contrast to this expression pattern, the characteristics observed in the CHI/VUE cohort were markedly different. CD3-positive T-lymphocytes were predominantly detectable in both compartments; however, CD4-positive T-lymphocytes were additionally identified in the villous space (21%) and the intervillous space (9%). Furthermore, 49% of all preparations exhibited CD8-positive T-lymphocytes present in both the intervillous and villous spaces. A significantly higher proportion of examined preparations also contained CD11c-positive cells (30%) (*p* = 0.03), which were predominantly localized in the intervillous space, often in conjunction with CD8-positive infiltrates. CD14-positive cells were detected in nearly all preparations across both compartments.

Notably, there were significant differences in the expression patterns of the markers CD68 and CD163. The detection of CD68 was not confined to the villous stroma, as it exhibited a ubiquitous distribution throughout the specimens, which was frequently observed in the intervillous space. Conversely, CD163 demonstrated a divergent distribution pattern from that observed in healthy specimens, with CD163-positive macrophages also found distributed ubiquitously.

The distribution patterns of the Coronavirus Disease 2019 (COVID-19) and COVID-19/CHI cohorts primarily adhered to one of the previously explained expression patterns. While the mere identification of maternal SARS-CoV-2 infection did not elicit any significant alterations in marker expression compared to the control group, the placentas from the CHI/COVID-19 cohort exhibited expression patterns akin to those observed in the CHI/VUE cohort. This suggests that SARS-CoV-2 infection does not manifest a distinct lesion pattern that diverges from that associated with chronic histiocytic intervillositis.

**Figure 2:**
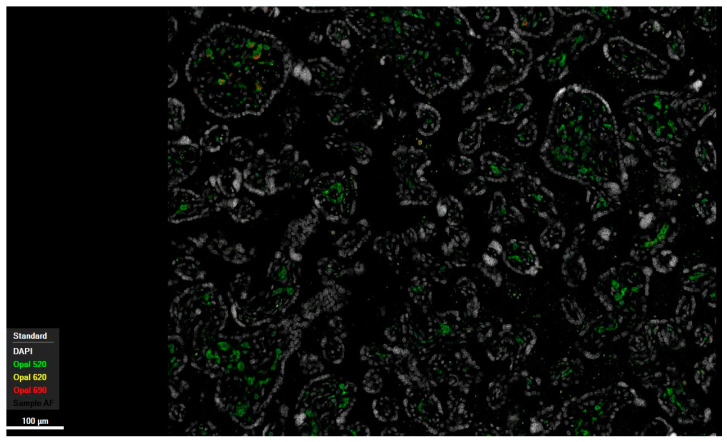
Multiplex immunofluorescence of a healthy human placenta (38 + 4 weeks of gestation). Opal 520 targeting CD163, Opal 620 targeting MHC II, and Opal 690 targeting CD68. Control group, 10× magnification.

**Figure 3:**
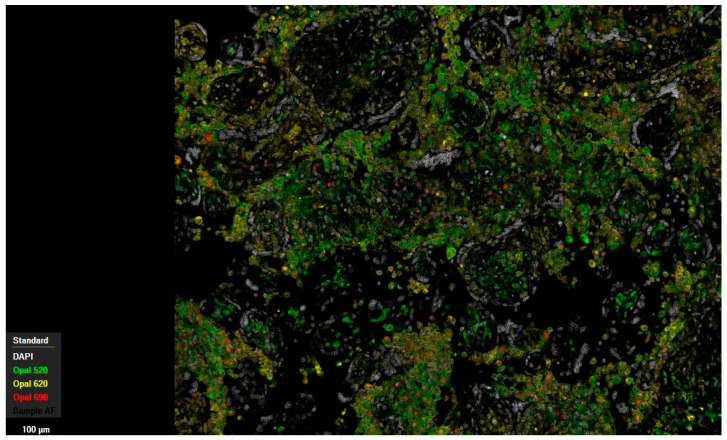
Multiplex immunofluorescence of a human placenta with chronic histiocytic intervillositis (31 + 6 weeks of gestation). Opal 520 targeting CD163, Opal 620 targeting MHC II, and Opal 690 targeting CD68; 10× magnification.

The analysis of macrophages for HLA-DR positivity, exemplarily performed on placentas exhibiting significant pathological changes (36 samples) and correlated with samples from the control group (10 samples), revealed substantial overexpression in maternal macrophages localized within the intervillous space. This was observed in both histopathologically unremarkable and inflammatory altered placentas, with a markedly stronger expression noted in the latter (Figure 2 and Figure 3). Additionally, a modest increase in expression of HLA-DR was also discernible within the chorionic villous stroma in inflammatory regions.

Multiplex immunofluorescence, in line with the above-described expression patterns of the surface antigens CD68 and CD163, showed qualitatively increased color intensity in inflammatory altered specimens in both compartments, fetal and maternal, compared to histopathologically unremarkable tissue, as did HLA-DR staining.

#### 2.2.2. Clinical Correlation of Marker Expression Strength Compared to Unremarkable vs. Remarkable Fetal Status

In further comparison, independent of the existing cohorts but strictly based on the same dataset as was represented in Table 1, an evaluation was made regarding the immunohistochemical expression of individual markers concerning the outcome of the respective pregnancies. For this, all placentas included in the study from pregnancies with confirmed intrauterine growth restriction of the fetus, severe fetal stress during delivery, or with at least one measurement of the APGAR score being less than or equal to 8 were selected. The inclusion of placentas into the remarkable fetal status cohort also allowed combinations of the above features.

The total number of included placentas was 60 in the group with an associated unremarkable fetal status (UFS) and 27 in the cohort with remarkable fetal status (RFS). As previously mentioned, attention was also given to the positivity of cells in two compartments, which resulted in each placenta being doubly included in the evaluation for differentiation, resulting in the total number of samples in the cohorts finally being 120 versus 54. In the evaluation, the detection of CD3-positive T-lymphocytes was 93.34% in the UFS cohort and 88.89% in the RFS. CD4 was only very sporadically observed in both cohorts, with percentages of 5.83% (UFS) and 5.55% (RFS), respectively. A notable increase in the number of CD8-positive T-lymphocytes was present in the RFS cohort, where positive samples amounted to 29.62%, while detection in the UFS cohort was only at 15.83%. Moreover, CD11c was more prevalent in the RFS group with 18.51% compared to 5.83% for the UFS cohort. Markers CD14-Magenta, CD68, and CD163 showed significant deviations (Table 1); the former was positively detected in 78.34% of all samples in the UFS group, while it was nearly ubiquitous in 96.29% of the samples in the RFS group. CD68 was detected in 60% of the UFS cohort compared to 90.75% in the RFS group, similar to CD163, which was detectable in 62.5% versus 81.49%.

These results largely correspond to those from the previous question; the expression pattern of placentas with unremarkable fetal status distinctly resembles that of control placentas (Table 2).

### 2.3. Statistical Evaluation

#### 2.3.1. Data Evaluation Regarding the First Question: Expression Strength of Investigated Immunohistochemical Markers in Fetal and Maternal Compartments Within Cohorts

**Figure 4:**
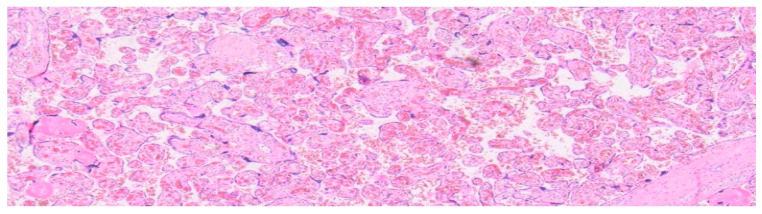
Light microscopy of a healthy human placenta (38 + 4 weeks of gestation) (hematoxylin and eosin staining; 4× magnification).

**Figure 5:**
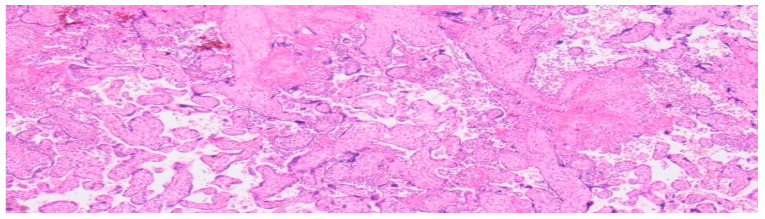
Light microscopy of a human placenta presenting with chronic histiocytic intervillositis (31 + 6 weeks of gestation) (hematoxylin and eosin staining; 4× magnification).

**Figure 6:**
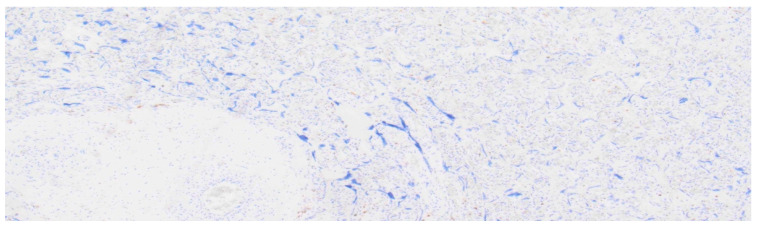
Detection of CD68-positive monocytes in healthy placental tissue; 4× magnification.

**Figure 7:**
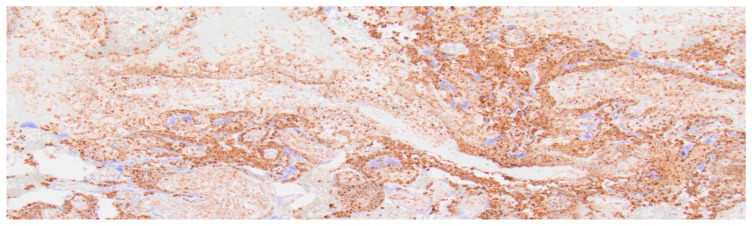
Detection of CD68-positive monocytes in a placenta with chronic histiocytic intervillositis; 4× magnification.

**Figure 8:**
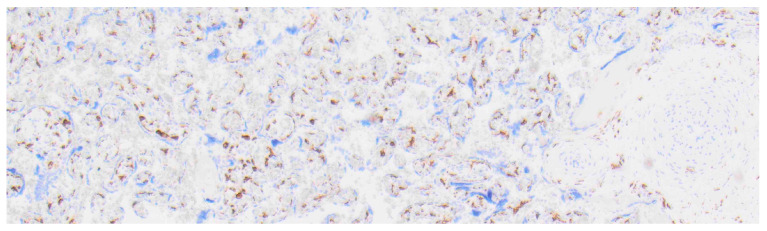
Detection of CD163-positive monocytes in healthy placental tissue; 4× magnification.

**Figure 9:**
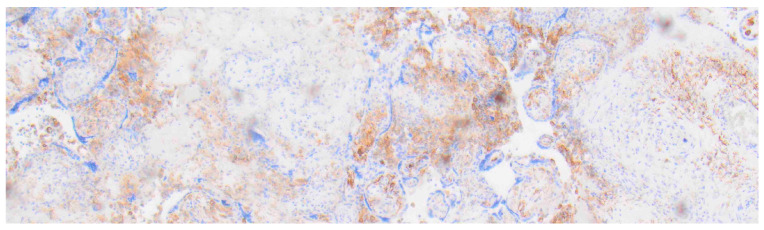
Detection of CD163-positive monocytes in a placenta with CHI; 4× magnification.

Following histopathological diagnosis, the results were first compared using contingency tables. This enabled the presentation of absolute and relative frequencies of the occurrence of individual cells positive for respective markers across the respective compartments and their isolated detection in the samples without regard to the localization of cells for comparison between cohorts (see Figure 1, Figure 2, Figure 3, Figure 4, Figure 5, Figure 6, Figure 7, Figure 8 and Figure 9). The contingency tables allowed for the calculation of *p*-values for the individual variables using Fisher’s exact test. The results of the first question, which isolated the cohorts and only distinguished the localization of positive cells for the markers, were illustrated using tables that provided detailed affirmations of the detection of positive cells in their respective compartments concerning the significance level.

In the statistical evaluation, the previously mentioned results were confirmed, demonstrating statistical significance at the *p* = 0.05 level regarding the distribution of positive cells for markers CD14, CD68, and CD163 within the control group, as well as in the COVID-19 cohort without CHI detection (Table 3). Positive cells here were found either predominantly or entirely isolated within one compartment. This distinct distribution pattern was lost in the presence of CHI or VUE and shifted towards pan-availability in both compartments, consistent with those in placentas of the CHI/COVID-19 cohort. Especially impressive was the visual result when stained with CD68 and CD163, as shown in Figure 6, Figure 7, Figure 8 and Figure 9.

#### 2.3.2. Comparison of Marker Expression Strength in Transcohort Analysis

**Figure 10:**
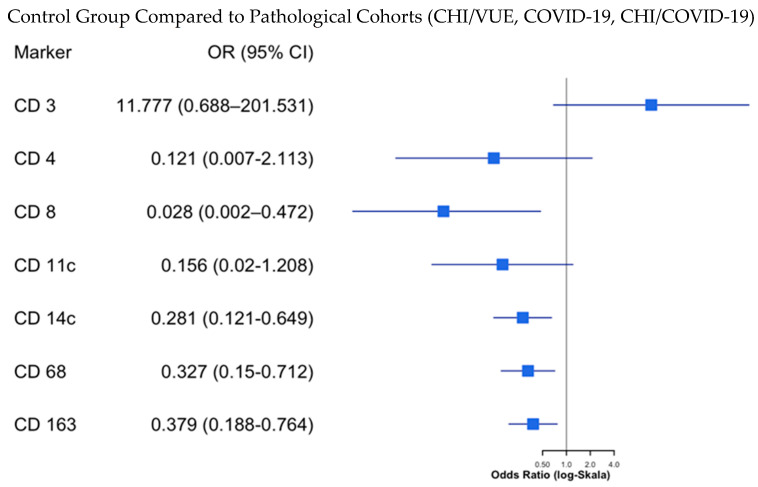
Forest plot showing confidence intervals and odds ratios for the comparison between the control group and pathological cohorts; *p* < 0.05.

**Figure 11:**
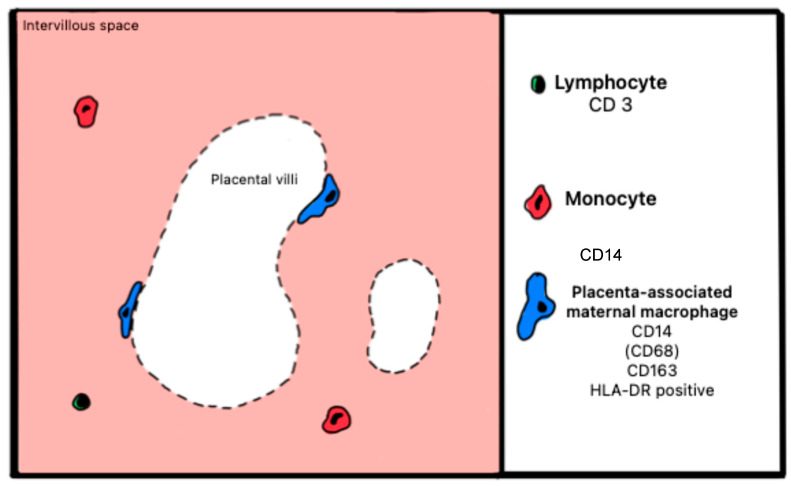
Schematic representation of cells expressing surface markers in the maternal intervillous space of a healthy placenta.

**Figure 12:**
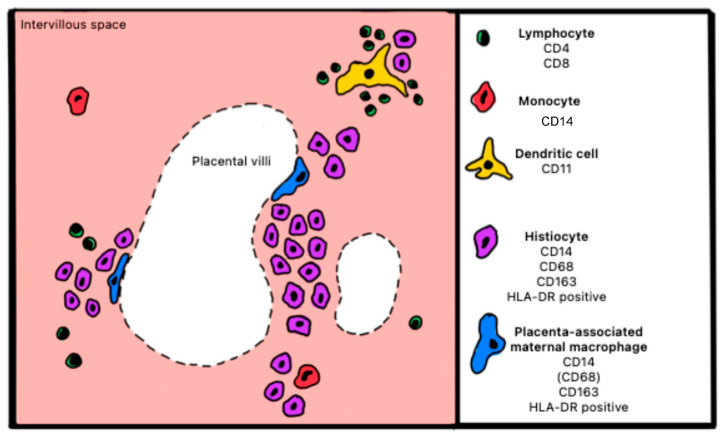
Schematic representation of cells expressing surface markers in the maternal intervillous space of a placenta with CHI.

**Figure A1:**
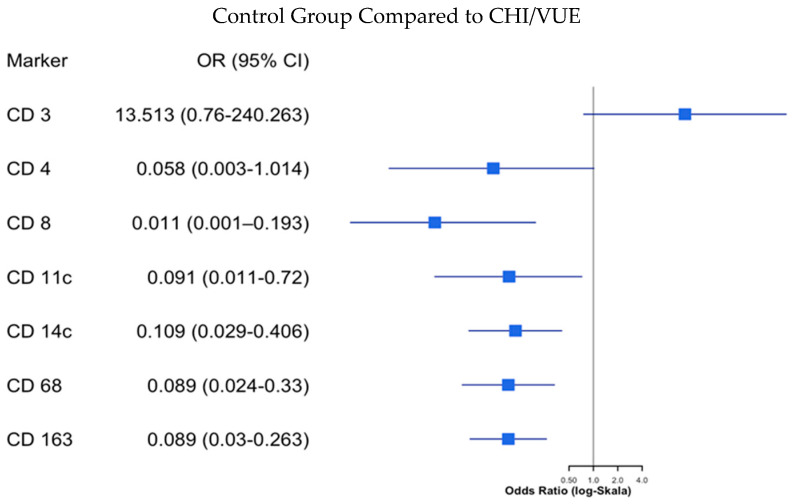
Forest plot showing confidence intervals and odds ratios for the comparison between the control group and CHI/VUE; *p* < 0.05.

**Figure A2:**
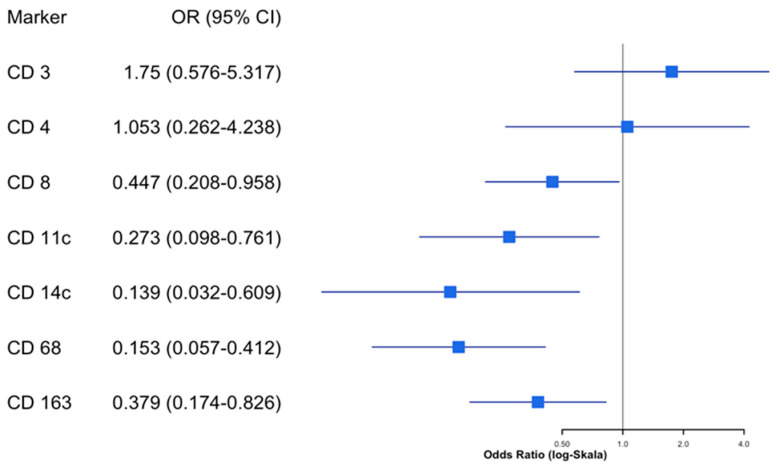
Forest plot showing the significant overexpression of individual markers in the comparison of abnormal versus normal fetal status.

The statistical evaluation of the variable “Marker Expression” was conducted based on contingency tables, utilizing odds ratios and confidence intervals derived from previously calculated *p*-values. Visualization was achieved through forest plots (Figure 10, Figure 11 and Figure 12). Due to the presence of zero values in the contingency tables, the Haldane correction was applied, which led to slightly distorted results. Despite this, significant *p*-values obtained through the exact Fisher test sometimes included one within the confidence interval. Nevertheless, this statistical analysis was performed in addition to previously applied methods to ensure clear visualization. A detailed comparison will focus on differences between the control group and pathological cohorts (CHI/VUE, COVID-19, CHI/COVID-19), with particular emphasis on the results comparing CHI/VUE against the control group (Figure 10). Additional visualizations can be found in Appendix A, Figure A1 and Figure A2. The findings are below.

The results indicate significant differences in the expression of markers in inflamed tissue, as proposed in the first question. Specifically, markers CD4, CD8, CD11c, CD14, CD68, and CD163 exhibit significantly increased expression in placentas with CHI and villitis of unclear etiology. In contrast, CD3, a marker detectable in both control and experimental placentas in native T-lymphocytes, shows no significant change in expression. No significant differences in expression were found in the cohort comparing control against COVID-19 without CHI, nor were there significant differences between CHI/COVID-19 and CHI/VUE. These results support the hypothesis that a maternal SARS-CoV-2 infection alone does not cause pathological changes in the placenta and that a maternal SARS-CoV-2 infection in conjunction with CHI does not lead to a distinct inflammatory profile separate from the characteristic findings of CHI. Furthermore, the fetal status showed a significant correlation between the antibody expression pattern of the immunohistochemical markers and fetal outcome (Appendix A).

## 3. Discussion

### 3.1. Presentation of Results

This study highlights the distinct inflammatory profiles associated with CHI and VUE. A significant overexpression of markers and an influx of activated immune cells were observed in placental compartments not typically evident under normal physiological conditions. Immunofluorescence analysis confirmed the heightened expression of CD68 and CD163, aligning with previous immunohistochemical findings. These pathognomonic changes in histopathology will be integrated with the current research landscape to provide nuanced insights [41,42,43,44,45,46,47,48,49,50,51,52,53,54,55,56,57,58].

Interestingly, this study also reveals that maternal SARS-CoV-2 infection alone does not significantly alter placental expression patterns. When combined with CHI, the inflammatory response mirrors that of CHI alone, suggesting that the viral infection does not show a unique inflammation pattern.

Moreover, the findings indicate a direct correlation between the increased presence of pro-inflammatory cells in the intervillous space and the incidence of intrauterine growth restriction, severe fetal distress, and reduced APGAR scores. This highlights the impact of placental inflammation on fetal health outcomes.

#### Atlas of Immune Cells at the Fetal–Maternal Interface

Figure 11 and Figure 12 illustrate healthy and pathological placenta immune cells at the interface within the maternal intervillous space.

**Figure 13:**
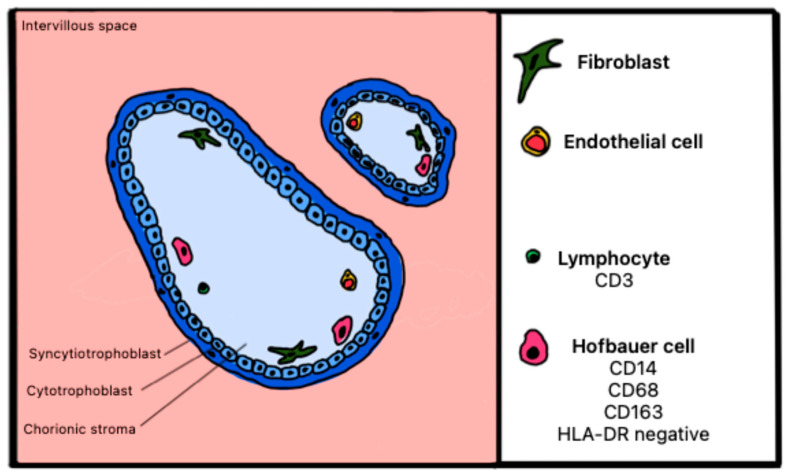
Schematic representation of cells expressing surface markers in fetal placental villi of a healthy placenta.

**Figure 14:**
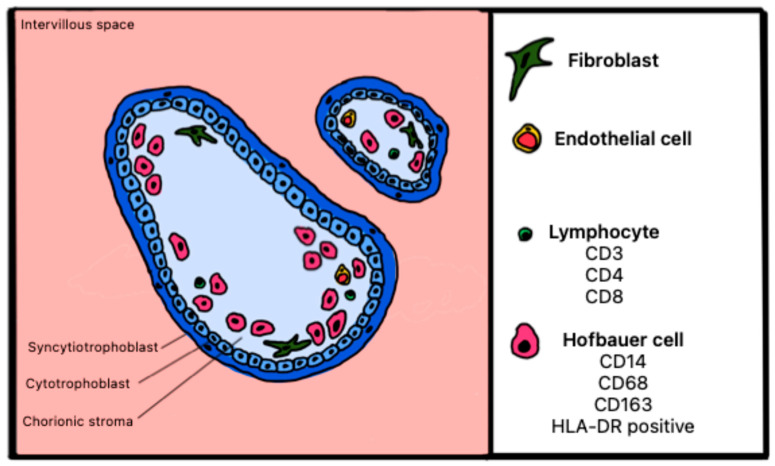
Schematic representation of cells expressing surface markers in fetal placental villi in a placenta with CHI.

Figure 13 and Figure 14 show healthy and pathological placenta immune cells at the interface within the fetal villous stroma.

### 3.2. Comparison with Current Study Findings

The analysis of positive marker specific - expression in the intervillous space and villous stroma reveals significant findings. In the control group, a ubiquitous presence of CD3-positive T-lymphocytes was noted, while CD4 and CD8 T-lymphocytes were undetectable. This aligns with the existing literature illustrating that CD3-positive lymphocytes dominate the placental immune landscape, comprising around 68% of the total lymphocyte population [59]. Variability in CD4 and CD8 detection was noted, with the presence of CD16-, CD20-, and CD56-positive cells contributing to the broader immune profile, though these were beyond the scope of this study [60].

The overwhelming presence of CD14-Magenta-positive monocytes in the villous stroma and their distribution within the intervillous space were consistent with observations in other studies, particularly the double expression of CD14 and CD68 in Hofbauer cells at the fetal–maternal interface [61]. CD68-positive monocytes were identified in both the intervillous space and villous stroma, with the expression of CD163 corroborating these findings [1].

Overall, our results align with the existing literature, setting a robust foundation for comparing expression patterns in control versus experimental cohorts. The specific histopathological alterations related to CHI and VUE, along with injury patterns associated with maternal SARS-CoV-2, will be elaborated upon in the following sections.

#### 3.2.1. Examination of Cell Image Morphology in Maternal SARS-CoV-2 Infection

The study distinctly noted that the presence of maternal SARS-CoV-2 infection did not yield unique histopathological changes compared to control samples. The expression patterns and cell distributions of marker-positive cells were effectively uniform across both cohorts. Some studies see a difference in CD163 expression in CHI versus CHI with SARS-CoV-2 infection [62]. While this study focused solely on placentas clinically linked to SARS-CoV-2, it is important to specify that no viral genome detection was performed in placental tissues, raising questions regarding viral implications for placental morphology.

#### 3.2.2. Expression Patterns in CHI and VUE

The current findings demonstrated a significant increase in CD4-, CD8-, CD11c-, CD14-Magenta-, CD68-, and CD163-positive cells, reinforcing findings from similar studies regarding the accumulation of CD68-positive histiocytes in inflammatory conditions [16]. It has been suggested that T-lymphocytes’ presence in the intervillous space increases in the face of CHI, contrasting with normal physiological states where regulatory T-cells tend to suppress these populations [15,51,52].

Notably, while CD68-positive histiocytes predominated in the inflammatory response, T-lymphocytes constituted up to 24% of the inflammatory cell populations. Their presence, along with specific CD4-positive regulatory T-cells, hints at a complex interaction that may suggest an autoimmune-mediated reaction toward the allogenic fetus [54].

The marked increase in antigen-presenting cells, indicated by CD11c staining, complements existing descriptions of infiltrating dendritic cells in the intervillous space [41]. Increased expression of histiocytic markers such as CD14-Magenta, CD68, and CD163 raises questions regarding the distribution of these histiocytes across various placental compartments, pointing to potential shifts in fetal macrophage polarization from M2 to M1 types [43]. Such polarization shifts, driven by cytokine interactions, may alter the activation profiles of Hofbauer cells, although this study did not specifically investigate associated markers like CD80, CD86, or CD40 [44].

Another possible explanation for the altered expression patterns could indeed be an infiltration of the villous space by maternal macrophages. For accurate differentiation, the study employed staining with HLA-DR in the context of multiplex visualization, as a marker that, while expressed by maternal macrophages, ranges from minimally to not expressed at all by fetal Hofbauer cells under physiological circumstances [54]. It should be noted that a shift in the polarization of Hofbauer cells during CHI may lead to increased expression of HLA-DR; however, this marker is the most sensitive for establishing differentiation [44,45]. This study found no clear infiltration of the villous stoma by maternal macrophages, consistent with further research in this area [55].

In keeping with the literature, this study shows the detection of large groups of CD68-positive histiocytes of maternal origin with infiltration of the intervillous space; this represents the most characteristic histopathological finding. Accompanying villous destruction, fibrinoid deposits, and vascular sclerosis are also typical findings [56].

The specific pattern of villitis of unknown etiology exhibits a perivillous emphasis on destruction, particularly mediated by CD3-, CD4-, and CD8-positive lymphocytes, alongside CD68-positive macrophages, with a focus on the primarily responsible CD8 T-lymphocytes. In this study, no isolated cases of villitis of unknown etiology were examined; rather, these were combined entities in a portion of the included placentas. All findings aligned with the current scientific consensus from Redline et al. on the lesion pattern of villitis of unknown etiology [16,46,47,48].

#### 3.2.3. Expression of Markers in CHI Associated with SARS-CoV-2

Clear, statistically significant patterns emerged regarding the inflammatory structures in placentas associated with maternal SARS-CoV-2 infection, mirroring those seen in isolated CHI. Although some studies have suggested potential viral replication in placental cells [42,58,63], this study found no distinctive lesion patterns differentiating SARS-CoV-2 from other RNA infections [39,40,58].

The heightened incidence of CHI in SARS-CoV-2 placentas [41,42,63] raises questions about the underlying mechanisms, suggesting that heightened awareness and research during the pandemic may have influenced detection rates, rather than indicating a direct causal relationship [57].

#### 3.2.4. Fetal Status in Relation to Marker Expression

The study proposed that an abnormal fetal status—characterized by intrauterine growth restriction, severe fetal stress, and an APGAR score ≤ 8—would lead to different expression patterns compared to the control group. This hypothesis was confirmed, with CD68 and CD163 exhibiting statistically significant changes in expression. Detailed statistical analysis and graphical representations are included in previous sections.

These results can be explained by diffusion restriction due to cell accumulation, combined with villous destruction and fibrinoid deposits in the intervillous space. Accumulated macrophages cause inflammation through the secretion of pro-inflammatory cytokines, further damaging placental tissue. This can impair the syncytiotrophoblast’s function and reduce the surface area available for nutrient exchange, contributing to fetal undernourishment. Additionally, cytokines can promote vascular modulation, resulting in hypoxic conditions and associated villous hypoplasia, disrupting placental blood flow. The interplay of mechanical factors, such as cell accumulation, fibrinoid deposits, and hemodynamic limitations, encapsulates the clinical impact on the fetus [49,50,53]. These findings are consistent with current research in the field.

### 3.3. Limitations of the Study

While this research offers valuable insights, several aspects warrant consideration. The cohort size, particularly within the CHI/COVID-19 group, was modest, with only three placentas included. Future studies could benefit from larger sample sizes to enhance the robustness of the findings, even though the current results still provide significant contributions to understanding the condition.

The study primarily focused on a specific set of immunohistochemical markers, which, while informative, present opportunities for future exploration into macrophage polarization and other potentially relevant markers that could expand our understanding of the immune response.

Additionally, the cohort comprised placentas from women at different stages of SARS-CoV-2 infection, which introduces variability in how infection timing might influence placental morphology. Although no PCR-based detection of the viral genome was performed, the findings still shed light on the relationship between maternal infection and placental pathology.

## 4. Materials and Methods

### 4.1. The Cohort

**Figure 15:**
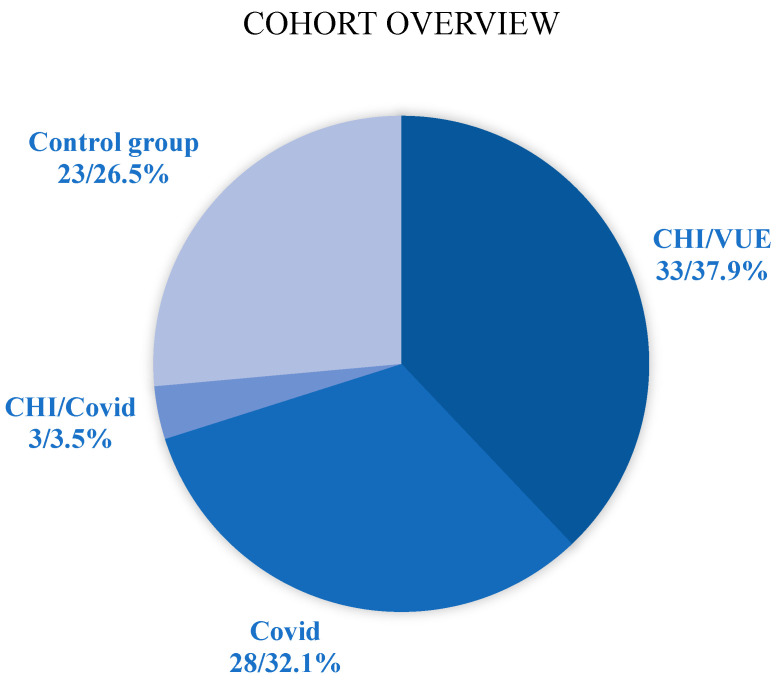
Pie chart showing the number of samples per cohort, including the experimental cohorts (CHI/VUE, COVID-19, CHI/COVID-19) and the control group.

**Figure 16:**
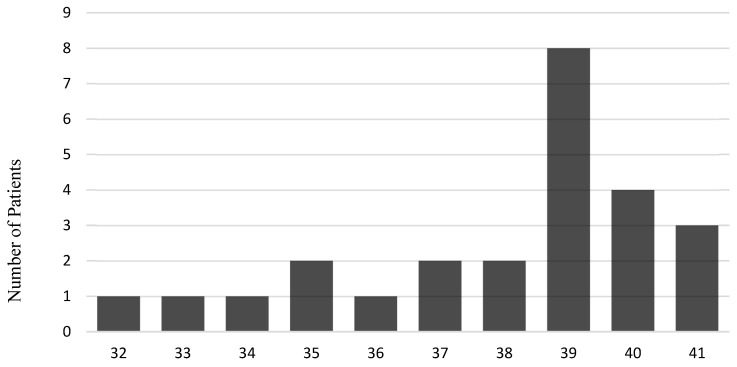
Gestational age distribution within the control group cohort.

**Figure 17:**
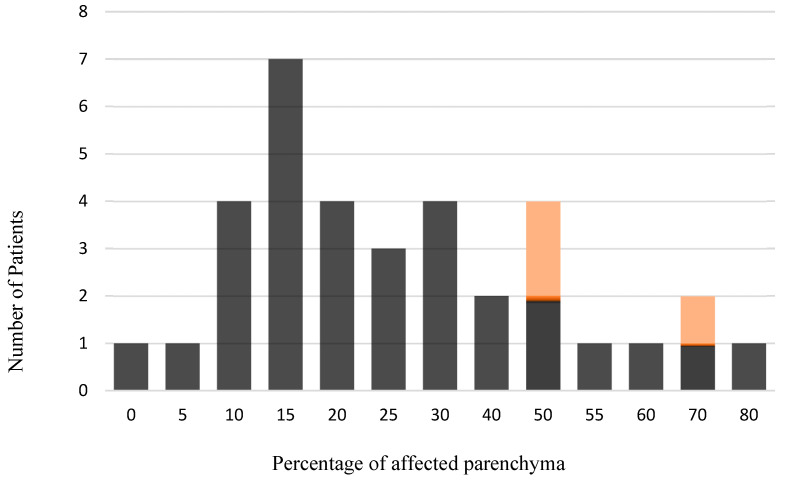
Percentage of parenchyma affected by inflammatory lesions in the CHI/VUE cohort, combined with the CHI/SARS-CoV-2 cohort (highlighted in orange).

The clinical data and histological samples were collected from placentas diagnosed between 2019 and 2024 at the Dr. Senckenberg Institute of Pathology, University Hospital Frankfurt am Main, Germany. The dataset includes 268 placentas with histological findings (for the distribution of histological diagnoses, see Figure 15), macroscopic descriptions of maternal and fetal surfaces, membrane conditions, and umbilical cord insertion types. Clinical information was taken from the placenta request form, which was completed by the obstetricians at the time of the delivery. This includes maternal age, number of pregnancies and births, BMI, gestational age (see Figure 16 and Figure 17), birth weight, APGAR score, and the indication for submission. Placentas were categorized into singleton and twin groups as “healthy” or “pathological.” Placentas were included in the “pathological” group if either CHI or CHI with VUE (CHI + VUE) was identified. Cases were excluded if they showed acute chorioamnionitis with maternal and fetal inflammatory response or isolated villitis with proven infection (other than COVID-19) or isolated VUE. “Healthy” placentas were included only when no pathology was observed histologically and no abnormal clinical information was reported.

The study received ethical approval from the Ethics Committee of the University Hospital Frankfurt (UCT-54-2020).

### 4.2. Histopathological Examination

Placenta assessments were conducted at the Dr. Senckenberg Institute of Pathology. This involved sectioning, embedding, and paraffin fixation of placentas, as well as preparing stained sections. For singleton placentas, four paraffin blocks were created; for twins, one block per twin plus two placenta blocks were prepared. HE-stained sections were reviewed to select the most suitable ones, which were stained with specific immunohistochemical markers. Histological sections were examined using a Carl Zeiss Axio Scope.A1 light microscope (MicroImaging GmbH, Oberkochen, Germany).

#### 4.2.1. Selection of Immunohistochemical Markers

For immunohistochemical phenotyping, various markers were used. To characterize the detected lymphocytic infiltrate in cases of CHI and VUE, the markers CD3 (Dako, Agilent, Glostrup, Denmark, antibody, F7.2.38) as an indicator of the presence of T-lymphocytes as part of the T-cell receptor (TCR) complex, as well as CD4 (Dako, Anti-Human, 4B12), a transmembrane glycoprotein particularly expressed by T-helper cells, and CD8 (Dako, Anti-Human, C8/144B), a membrane protein that acts as a co-receptor of the TCR and serves to recognize MHC-I molecules and is expressed by cytotoxic T-cells, were used. Additionally, CD11c (Leica Biosystems, Nussloch GmbH, Nussloch, Germany, Anti-Human, 5D11), a marker expressed by monocytes, macrophages, and dendritic cells, as well as CD14 (DCS Inn, Hamburg, Germany,, antibody, EPR3653), a glycoprotein also expressed by macrophages and monocytes, were employed. Classical histiocytic markers CD68 (Dako, Anti-Human, KP1) and CD163 (Cell Marque, Rocklin, CA, USA, antibody, MRQ-26) were also used. To differentiate between maternal macrophages and Hofbauer cells, one section each from the CHI and CHI/COVID-19 groups was stained with HLA-DR (Abcam, Cambridge, UK, Anti-Human, ERP3692), a receptor that is consistently expressed by maternal macrophages but very minimally by Hofbauer cells.

#### 4.2.2. Immunohistochemical Stains

The subsequent evaluation of the stained sections included the detection of positive cells for the respective immunohistochemical antibodies in two compartments: the maternal intervillous space and the fetal chorionic villous stroma. A finding was deemed positive as soon as one positive cell was detectable. Additionally, the percentage of placental areas affected by inflammatory infiltrate was assessed. The evaluation was then conducted dichotomously, separating into positive and negative.

The primary focus of the staining conducted here was on the detection and differentiation of lymphocytes and monocytes in the intervillous space and the villous stroma. The histological images of each marker are presented, comparing the healthy control group to CHI (see Figure 4, Figure 5, Figure 6, Figure 7, Figure 8 and Figure 9).

### 4.3. Multiplex Immunofluorescence

To achieve precise detection of specific macrophage populations and their spatial relationship with target structures, multiplex immunofluorescence was performed using specific antibodies against the surface antigens CD68, CD163, and MHC II. Two control placentas and three placentas from the CHI/VUE group were selected and processed as representative examples. The required histological sections were obtained from the same blocks from which the previously prepared sections for immunohistochemical staining were derived, allowing for direct comparison of identical tissue sections.

**Figure A3:**
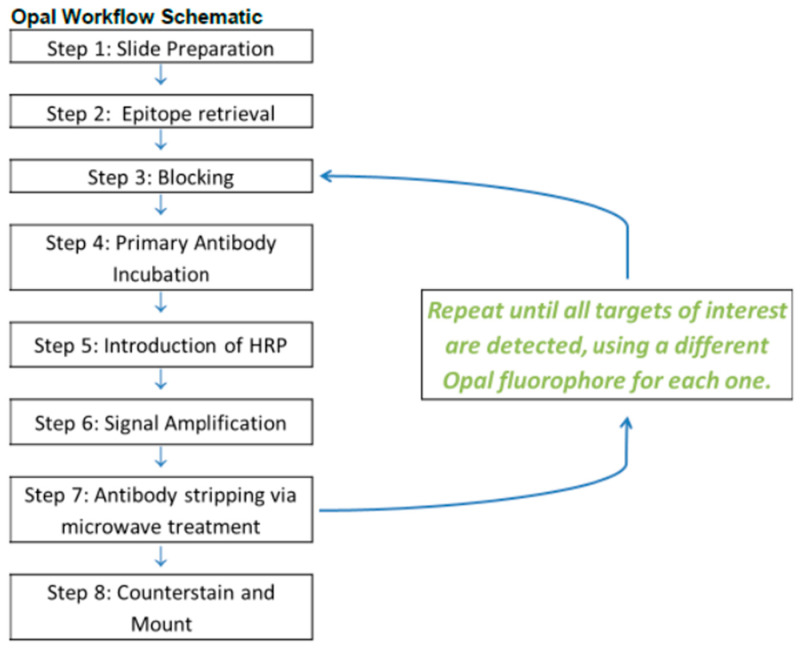
Flowchart of the multiplex immunofluorescence procedure.

Following incubation with antibodies targeting the aforementioned antigens, secondary HRP-conjugated antibodies and Opal fluorophores were applied for optical detection. The following fluorescent dyes were utilized: Opal 520 for CD163, Opal 620 for MHC II, and Opal 690 for CD68. Detailed staining protocols can be found in the Appendix B (Figure A3). For general nuclear staining, 4′,6-diamidino-2-phenylindole (DAPI) was employed, typically visualized in blue; however, this study aimed for a depiction in white to enhance contrast. Further details of the applied methods, including details of the antibody clones, can be found in Appendix B (Table A1).

### 4.4. Data Collection and Statistical Analysis

Four cohorts were created based on clinical and histopathological data: one control (unremarkable placentas) and three experimental groups—pathological placentas with confirmed CHI and VUE, placentas from mothers with SARS-CoV-2 infections, and an overlap containing placentas exhibiting both CHI and SARS-CoV-2.

All included placentas ranged from 24 + 6 to 41 + 6 weeks of gestation, with controls matched accordingly. Placentas with inflammatory changes were selected after excluding infectious backgrounds and advanced autolysis. Control placentas were selected based on the absence of placental-associated pathologies. Data were gathered for several research questions, examining the placenta in two compartments: fetal chorionic villous stroma and maternal intervillous space. Positively marked cells were compared within and between groups.

Subsequently, the expression strength of individual markers was examined among all groups. A differentiation between Hofbauer cells and maternal macrophages was also performed within the CHI and CHI/COVID-19 groups.

Data were presented in contingency tables and analyzed using Fisher’s exact test (*p* = 0.05). Odds ratios and confidence intervals were calculated, with visualizations via forest plots for further analysis. Statistical evaluation was conducted using the open-source program R (R 4.4.3).

## 5. Conclusions

CHI is a rarely diagnosed yet clinically significant condition. This diagnosis is established solely through histological examination, which is not routinely performed for placentas of pregnancies with abnormal fetal outcomes in Germany [64]. It is crucial for affected women and warrants further research, closer monitoring of subsequent pregnancies, and potentially standardized therapy. Although this study did not address treatment options for this condition or villitis of unknown etiology, it aims to enhance the recognition of both lesions associated with CHI/VUE by histopathologists without specific perinatal expertise, utilizing the atlas-like presentation of the findings.

The results demonstrate that CD68 serves as a valuable marker for the straightforward detection of CHI in general histopathology laboratories. While the presence of CD68-positive macrophages in this condition is well-documented, the statistically significant results of this study affirm its capability to detect this specific lesion. By ruling out an infectious origin clinically, CD68 staining provides a clear visual pattern that minimizes the risk of overlooking the condition. This enhances diagnostic accuracy and enables appropriate monitoring for affected women in future pregnancies due to the high risk of recurrence.

## Figures and Tables

**Table 1:** Overview of the number of samples demonstrating a positive detection of specific immune markers across different cohorts (CHI/VUE, COVID-19, COVID-19/CHI, and control) in the specified compartments (villous stroma and intervillous stroma).

Antibody	Compartment	CHI/VUE	COVID-19	COVID-19 with CHI	Control
CD3	Villous stroma	31/97%	24/86%	3/100%	23/100%
	Intervillous stroma	27/82%	26/93%	3/100%	23/100%
CD4	Villous stroma	7/21%	0/0%	0/0%	0/0%
	Intervillous stroma	3/9%	0/0%	0/0%	0/0%
CD8	Villous stroma	16/49%	0/0%	1/33%	0/0%
	Intervillous stroma	16/49%	0/0%	2/67%	0/0%
CD11c	Villous stroma	3/9%	0/0%	0/0%	0/0%
	Intervillous stroma	10/30%	2/7%	1/33%	1/4%
CD14	Villous stroma	33/100%	28/100%	3/100%	23/100%
	Intervillous stroma	30/91%	17/61%	3/100%	9/39%
CD68	Villous stroma	30/91%	28/100%	3/100%	23/100%
	Intervillous stroma	33/100%	12/43%	3/100%	7/30%
CD163	Villous stroma	33/100%	28/100%	3/100%	23/100%
	Intervillous stroma	28/85%	0/0%	3/100%	1/4%

**Table 2:** Overview of positive immunohistochemical expression by marker in relation to fetal status.

Marker	UFS (120 Samples)	RFS (54 Samples)
CD3	112/93%	48/89%
CD4	7/5.8%	3/6%
CD8	19/15.8%	16/30%
CD11c	7/5.8%	10/19%
CD14m	94/78.3%	52/96%
CD68	72/60%	49/91%
CD163	75/62.5%	44/81%

**Table 3:** Statistical correlation of the cohorts and distribution of positive cells across compartments. “Significance” indicates statistically significant results with asterisks: * (*p* < 0.05), and *** (*p* < 0.001).

Marker	Cohort	Villous Space-Positive	Intervillous Space-Positive	Odds Ratio	95% CI (Lower)	95% CI (Upper)	*p*-Value	Significance
**CD3**	CHI/VUE	31	27	2 98	0.63	13.97	0.152	
	COVID-19	24	26	0.51	0.10	2. 65	0.420	
	CHI/COVID-19	3	3	1.00	0.02	66.06	1.000	
	Control Group	23	23	1.00	0.02	52.53	1.000	
**CD4**	CHI/VUE	7	3	2.47	0.63	9.72	0.188	
	COVID-19	0	0	1.00	0.02	52.15	1.000	
	CHI/COVID-19	0	0	1.00	0.02	66.06	1.000	
	Control Group	0	0	1.00	0.02	52.53	1.000	
**CD8**	CHI/VUE	16	16	1.00	0.39	2.59	1.000	
	COVID-19	0	0	1.00	0.02	50.89	1.000	
	CHI/COVID-19	1	2	0.36	0.02	6.30	0.480	
	Control Group	0	0	1.00	0.02	52.53	1.000	
**CD11c**	CHI/VUE	3	10	0.26	0.07	0.97	0.036	*
	COVID-19	0	2	0.19	0.01	4.05	0.236	
	CHI/COVID-19	0	1	0.24	0.01	8.62	0.414	
	Control Group	0	1	0.32	0.01	8.25	0.470	
**CD14**	CHI/VUE	33	30	7.69	0.38	154.97	0.122	
	COVID-19	28	17	37.46	2. 07	676.21	<0.001	***
	CHI/COVID-19	3	3	1.00	0.02	66.06	1.000	
	Control Group	23	9	71.74	3.88	1327.65	<0.001	***
**CD68**	CHI/VUE	30	33	0.13	0.01	2.62	0.122	
	COVID-19	28	12	75.24	4.18	1355.16	<0.001	***
	CHI/COVID-19	3	3	1.00	0.02	66.06	1.000	
	Control Group	23	7	103.40	5.52	1938.58	<0.001	***
**CD163**	CHI/VUE	33	28	12.93	0.69	244.05	0.032	*
	COVID-19	28	0	3249.00	62.30	169,442.06	<0.001	***
	CHI/COVID-19	3	3	1.00	0.02	66.06	1.000	
	Control Group	23	1	705.00	27.27	18,225.83	<0.001	***

## Data Availability

All datasets will be available upon request.

## Abbreviations

The following abbreviations are used in this manuscript:

CHI	Chronic Histiocytic Intervillositis
HBCs	Hofbauer Cells
PAMMs	Placenta-Associated Maternal Macrophages
HLA	Human Leukocyte Antigen
VUE	Villitis of Unknown Etiology
CD	Cluster of Differentiation
TGFBR1	Transforming Growth Factor Beta Receptor 1
MMP	Matrix Metalloproteinase
SARS-CoV-2	Severe Acute Respiratory Syndrome Coronavirus 2
APGAR	Appearance, Pulse, Grimace, Activity, Respiration (scoring system)
HE	Hematoxylin and Eosin (staining)
DAPI	4′,6-Diamidino-2-phenylindole (nuclear stain)
HRP	Horseradish Peroxidase
CI	Confidence Interval
UFS	Unremarkable Fetal Status
RFS	Abnormal Fetal Status

## Appendix B. Detailed Methods for the Multiplex Immunofluorescence

All placenta sections were stained with Opal Multiplex IHC Kit (Akoya Bioscience, Marlborough, MA, USA) in the BOND-RX Multiplex IHC Stainer (Leica, Wetzlar). The primary antibodies used were anti-CD163 (abcam, ab182422), anti-MHCII (Cell Signaling, 68258S), and anti-CD68 (DAKO, M087601-2), as well as Opal Anti-Ms + Rb HRP (ARH1001EA, Akoya Bioscience) as a secondary antibody in sequential staining. Nuclei were counterstained with 4′,6-diamidino-2- phenylindole (DAPI, FP1490, Akoya Bioscience) and mounted with Fluoromount-G (SouthernBiotech, Birmingham, AL, USA). To test the epitope stability of all targets, tissue sections were repeatedly subjected to heat-induced antigen retrieval, and changes in antigen-dependent signal after staining were monitored. The order of antibody staining was selected based on this validation process. A Polaris imaging system (Akoya Bioscience) was used to image the slides at 20x magnification.

**Table A1:** Antibody clones with dilution.

	AK	Clone		Dilution	Cooking Buffer	Host	Opal
1	CD163	PG-M1	ab182422; abcam	250	pH9	rabbit	520
2	MHCII	LGII-612.14	68258S; Cell Signaling Technology	400	pH9	mouse	620
3	CD68	EPR19518	M087601-2; DAKO	100	pH9	mouse	690
